# Serotonin Syndrome Triggered by an Interaction Between Opioid Analgesics and Cobicistat: A Challenging Diagnostic Case Report

**DOI:** 10.7759/cureus.47138

**Published:** 2023-10-16

**Authors:** Pareena Sharma, Reid W Collis, Julien Thomas

**Affiliations:** 1 Department of Neurology, Medical College of Georgia at Augusta University, Augusta, USA; 2 Department of Physical Medicine and Rehabilitation, Wellstar Kennestone Hospital, Marietta, USA; 3 Department of Neurology, Wellstar Kennestone Hospital, Marietta, USA

**Keywords:** immunocompromised, oxycodone, symtuza, cobicistat, serotonin syndrome diagnosis

## Abstract

Serotonin syndrome is a clinically diagnosed disorder that may occur secondary to medications that increase the release of endogenous serotonin, impair the reuptake of serotonin from the synaptic cleft, are direct serotonin receptor agonists, or increase the sensitivity of the postsynaptic serotonin receptor. In this case report, we describe the diagnosis of serotonin syndrome in a 60-year-old immunocompromised male. This case is unique, as many of the medications associated with the development of serotonin syndrome in this patient are not typically thought of as being associated with serotonin syndrome, though, in this clinical context, they combined to produce a profound pro-serotonergic effect.

## Introduction

Serotonin syndrome (SS) describes a clinically diagnosed and potentially life-threatening drug interaction involving serotonergic agents. SS is considered a rare disease, as mild cases are often overlooked, and severe cases are often attributed to other causes [1]. SS is commonly caused by the ingestion of selective serotonin reuptake inhibitors (SSRIs), serotonin and norepinephrine reuptake inhibitors (SNRIs), and monoamine oxidase inhibitors (MAOIs). Studies show that MAOIs have the highest risk for developing severe SS, with SS due to SNRIs and SSRIs being less common [2]. The most widely recognized case of SS was of an 18-year-old woman who is thought to have suffered from severe SS due to meperidine, an opiate, and phenelzine, an MAOI, among other serotonergic medications that led to her death within eight hours of admission in 1984. As demonstrated by this historic case, SS can be rapidly fatal. SS is also associated with complications such as rhabdomyolysis, respiratory failure, and coma [3].

The Hunter Serotonin Toxicity Criteria are a group of clinical features associated with serotonin syndrome that are highly sensitive and specific in the diagnosis of the condition. To meet the Hunter Criteria for SS, a patient needs to 1) be on a serotonergic agent and 2) present with one or more of clonus, hypertonia, and hyperthermia, or tremor and hyperreflexia [4]. Some have argued that these criteria are too subjective and require more concrete, objective measures for ruling in SS more effectively [5]. Moreover, SS can also be induced by a variety of other serotonergic agents, some of which are less commonly discussed and can be overlooked when raising suspicion for SS. In this report, we discuss a case of SS that involved fentanyl, venlafaxine, fluconazole, meperidine, and a rare interaction with oxycodone and an HIV combination regimen of darunavir, cobicistat, emtricitabine, and tenofovir alafenamide.

## Case presentation

**Figure 1:**
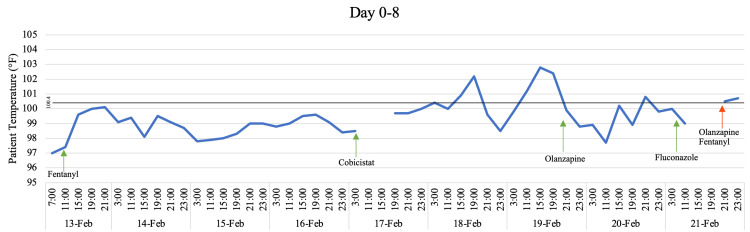
Temperature trend on Days 0 through 8 with inclusion of key medications Green arrows are indicative of a medication's first administration. Red arrows are indicative of a medication's final administration. Cobicistat is listed as a placeholder for the four-drug combination of darunavir, cobicistat, emtricitabine, and tenofovir alafenamide.

A 60-year-old male with a medical history of controlled HIV treated with a four-drug, fixed-dose combination product of darunavir, cobicistat, emtricitabine, and tenofovir alafenamide, non-Hodgkin's lymphoma in remission, and depression treated with venlafaxine, presented to the emergency department with altered mental status, lethargy, and respiratory distress. He was intubated with a combination of etomidate, rocuronium, versed, and fentanyl, and was placed on fentanyl and propofol drips for sedation (Figure 1). Neurological symptoms included intermittent responsiveness and somnolence on the initial neurology exam.

Lab results showed urine toxicology positive for oxycodone and chemistry panel concerning for metabolic acidosis. Chest CT and bronchoscopy revealed bilateral lower lobe purulence suggestive of aspiration pneumonia, and treatment with cefepime, metronidazole, and vancomycin was initiated by the Infectious Disease service.

On the fourth day, the patient resumed his HIV combination product of darunavir, cobicistat, emtricitabine, and tenofovir alafenamide and subsequently developed a fever with a maximum temperature of 102.2F (Figure 1). The antibiotics were broadened to meropenem and vancomycin due to continued fever. Extensive testing including multiple CT scans of the chest, abdomen, and pelvis, as well as multiple blood, tracheal aspirate, bronchoalveolar lavage, urine, and cerebrospinal fluid cultures for infectious sources yielded no evidence of infection. Additionally, cerebrospinal fluid drawn more than two weeks apart showed a normal white blood cell count and protein level and only one sample with mildly elevated glucose.

On Day 6, the patient was started on olanzapine to aid in weaning off sedation (Figure 1). On day seven, the patient began opening his eyes intermittently but was unresponsive to commands. The Infectious Disease service was consulted again on Day 8 for a fungal neck rash, and the patient was started on fluconazole (Figure 1). Given continued decreased responsiveness, the patient was taken off both olanzapine and the fentanyl drip, which had previously been precluded by profound tachypnea with decreasing sedation (Figure 1).

On Day 9, the patient was noted to have significant hyperreflexia in their bilateral upper and lower extremities, with positive Hoffman’s sign but no lead pipe rigidity. Whole spine MRI was performed, which was negative for any acute abnormalities.

**Figure 2:**
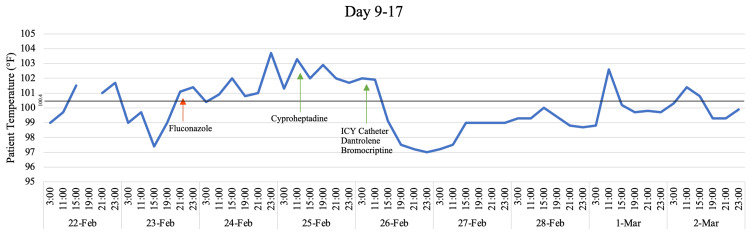
Temperature Trend on Days 9 Through 17 With Inclusion of Key Medications Green arrows are indicative of a medication's first administration. Red arrows are indicative of a medication's final administration.

On Day 10, the patient's condition worsened, and he was observed to be persistently hyperthermic, hyperreflexic, and tremulous without rigidity. Concerns for possible serotonin syndrome led to discontinuation of fluconazole and initiation of cyproheptadine. Further deterioration was observed, and the patient exhibited bilateral positive clonus. An evening dose of meperidine was given to manage excessive shivering caused by persistent hyperthermia between 101F and 104F, which was additionally managed using an ICY catheter (Figure 2). The patient continued to have hyperreflexia with a positive Hoffman's sign and bilateral positive clonus. Subsequent brain MRI showed no acute changes.

**Figure 3:**
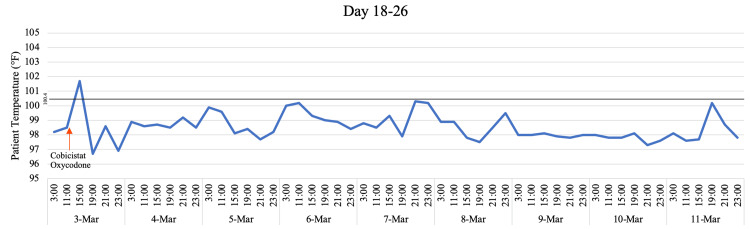
Temperature Trend on Days 18 Through 26 With Inclusion of Key Medications The red arrow is indicative of a medication's final administration. Cobicistat is listed as a placeholder for the four-drug combination of darunavir, cobicistat, emtricitabine, and tenofovir alafenamide.

Due to the lack of improvement with cyproheptadine and continued hyperthermia after fentanyl had been stopped, dantrolene and bromocriptine were started, which preceded some improvement with mildly increased responsiveness and reduced hyperreflexia (Figure 2). However, recovery plateaued until all medications with any pro-serotonergic activity, including cobicistat, oxycodone, and hydromorphone were ceased six days later. Within 24 hours after cessation of these medications, the patient showed significant improvement in mental status, hyperreflexia, and subsequent defervescence, eventually returning to his neurologic baseline (Figure 3).

## Discussion

This case describes a patient who developed severe serotonin syndrome involving fentanyl, oxycodone, venlafaxine, meperidine, and fluconazole, in combination with the CYP2D6 and CYP3A4 inhibitor cobicistat [6]. While cobicistat does not directly increase serotonin levels, its combination with other serotonergic medications, such as fentanyl, oxycodone, hydromorphone, and venlafaxine, which are all metabolized by one or both enzymes inhibited by cobicistat [7-8], may have led to persistent serotonergic activity. Though oxycodone is not typically thought of as a causative agent for severe serotonin syndrome, with an unclear mechanism of serotonergic effect, some studies suggest that a direct serotonin receptor agonism (Table 1) in the setting of co-administration of other serotonergic medications may amplify its effects [7,9].

**Table 1:** Comprehensive List of Serotonergic Agents and Associated Mechanisms Starred terms indicate medications and classes that this patient was exposed to prior to or during the development of the presumed serotonin syndrome. MDMA: 3,4-Methyl​enedioxy​methamphetamine; MAO: monoamine oxidase;

Mechanism	Agent
Increases serotonin formation	Tryptophan. oxitriptan
Increases release of serotonin	Amphetamines (including dextroamphetamine and methamphetamine)
	MDMA (ecstasy)
	Amphetamine derivatives (including fenfluramine, dexfenfluramine, phentermine)
	Cocaine
	Mirtazapine
Impairs serotonin reuptake from the synaptic cleft into the presynaptic neuron	Cocaine
	MDMA (ecstasy)
	*Meperidine
	Tramadol
	Pentazocine
	Dextromethorphan
	Selective serotonin reuptake inhibitors
	*Serotonin-norepinephrine reuptake inhibitors
	Sibutramine
	Bupropion
	Serotonin modulators (nefazodone, trazodone, vilazodone, and vortioxetine)
	Cyclic antidepressants
	St. John's wort (Hypericum perforatum)
	5-HT3 receptor antagonists (dolasetron, granisetron, ondansetron, palonosetron)
	Cyclobenzaprine
	Methylphenidate, dexmethylphenidate
Nonselective and selective MAO-A and B inhibitors	MAO inhibitors, nonselective
	MAO-A inhibitors
	MAO-B inhibitors
Direct serotonin receptor agonist	Buspirone
	Triptans
	Ergot derivatives
	*Fentanyl
	Lysergic acid diethylamide (LSD)
	Lasmiditan
	Lorcaserin
	Metaxalone
	*Oxycodone
Increases sensitivity of postsynaptic serotonin receptor	Lithium
Decreases metabolism of serotonergic agents	*Fluconazole (CYP2C9 and CYP3A4 inhibitor)
	*Cobicistat (CYP2D6 and CYP3A4 inhibitor)

The true incidence of serotonin syndrome is difficult to ascertain, as cases are likely underreported [10]. However, the Toxic Exposure Surveillance System indicates that approximately 15% of SSRI poisoning cases meet the criteria for serotonin syndrome [10]. Additionally, a retrospective study conducted in 2017 suggests that the overall incidence of serotonin syndrome falls within the range of 0.19% to 0.07%, making it a rare condition [10].

While the early signs of hyperthermia started after the re-administration of the four-drug fixed-dose combination product of darunavir, cobicistat, emtricitabine, and tenofovir alafenamide, it was unclear whether the patient had SS at that time, particularly in the setting of their initial presentation of aspiration pneumonitis. The Infectious Disease service was consulted and found no subsequent infectious cause for the hyperthermia, despite a thorough workup. With non-inflammatory cerebrospinal fluid on two separate occasions, as well as negative antibody testing for a range of inflammatory diseases, auto-immune pathologies were thought to be unlikely. Though SS entered the differential upon the appreciation of hyperreflexia on physical exam, neuroleptic malignant syndrome (NMS) presented a second possibility in this patient given previous olanzapine administration and the patient's hyperthermia. The purely clinical and vague criteria for SS overlap with other syndromes like NMS and malignant hyperthermia. Specifically, both SS and NMS present similarly with altered mental status, hyperthermia, and hypertonia [3]. However, NMS is characterized by hyporeflexia and profound hypertonia manifesting as rigidity while SS exhibits milder hypertonia and significant hyperreflexia [3]. Identifying milder cases or early warning signs of SS and NMS may be difficult to differentiate. The confluence of these exam findings complicated this patient’s clinical course and rendered the ultimate diagnosis of serotonin syndrome challenging until the presentation became more severe.

Combination medication regimens, including cobicistat, may be associated with an increased risk for serotonin syndrome given many serotonergic agents are metabolized by CYP2D6 and CYP3A4 (Table 2) [11].

**Table 2:** Serotonergic Agents That Are Metabolized by CYP2D6 and/or CYP3A4 MDMA: 3,4-Methyl​enedioxy​methamphetamine; SSRI: selective serotonin reuptake inhibitor; SNRI: serotonin and norepinephrine reuptake inhibitor

Metabolizing Enzyme	Serotonergic Agent
CYP2D6 and CYP3A4	Oxycodone
	Amphetamine Derivatives
	Mirtazapine
	Tramadol
	Ondansetron
	Palonosetron
	Cyclobenzaprine
	Lorcaserin
	Metaxalone
CYP2D6	Amphetamines
	Lithium
	MDMA
	Dextromethorphan
	SSRIs
	SNRIs
	Vortioxetine
	Dolasetron
	Cyclic antidepressants
CYP3A4	Fentanyl
	Cocaine
	Meperidine
	Nefazodone
	Trazodone
	Vilazodone
	St. Johns Wart
	Buspirone
	Ergot derivatives

Similar interactions have been noted in previous literature, including a 2012 case report where a patient on HIV medication darunavir/ritonavir, proton pump inhibitor esomeprazole, and SSRI escitalopram developed a similar case of SS [12], and a 2019 case involving a patient on an antiretroviral therapy of elvitegravir, cobicistat, emtricitabine, and tenofovir disoproxil fumarate who was started on escitalopram for anxiety, with concomitant receipt of ondansetron for nausea, who developed serotonin syndrome [13]. While it is well known that CYP inhibitors have the potential to cause SS, patients taking HIV combination antivirals with CYP2D6 and CYP3A4 inhibitors may be at higher risk of being underdiagnosed given the abundant drug interactions involved in HIV treatment regiments.

## Conclusions

It is difficult to make a diagnosis of SS because there are numerous potential causative agents, the criteria are purely clinical, and the syndrome itself is rare. However, this case calls for attention to the expansive list of agents that should raise alarm for SS, including CYP2D6 and CYP3A4 inhibitors, as well as more specific criteria for diagnosing and staging SS. In particular, special attention to combination medication regimens, including cobicistat, along with oxycodone, which is not typically associated with SS, may help prevent the propagation of this already underdiagnosed syndrome.
